# Operative experience in paediatric orthopaedics in UK trainees achieving a Certificate of Completion of Training in trauma and orthopaedic surgery: A descriptive analysis of national eLogbook data

**DOI:** 10.1308/rcsann.2025.0057

**Published:** 2025-08-29

**Authors:** T Barrow, BD Chatterton, SN Maripuri, T Crompton, N Kiely, K James

**Affiliations:** ^1^ University Hospitals Sussex NHS Foundation Trust, UK; ^2^ Robert Jones and Agnes Hunt Orthopaedic Hospital NHS Foundation Trust, UK

**Keywords:** Orthopaedics, Trauma, Paediatrics, Training, Education, Workforce planning

## Abstract

**Introduction:**

The Trauma and Orthopaedic curriculum set by the Joint Committee on Surgical Training (JCST) requires that consultant orthopaedic surgeons must have sufficient experience managing children’s orthopaedic conditions. In this study, our objective was to describe the paediatric operative exposure of United Kingdom (UK)-trained orthopaedic registrars who obtain a Certificate of Completion of Training (CCT).

**Methods:**

This was a national retrospective cohort study of UK trainees obtaining a CCT between 1 January 2018 and 1 January 2024. ELogbook data for 884 trainees were obtained from the JCST, with 1,994,235 recorded operations. Descriptive analysis was performed on the operative data.

**Results:**

Our results demonstrated that the median number of cases per trainee throughout training decreased each year across both adult and paediatric experience. The proportion of paediatric cases (age <17 years) within the trainees’ logbooks remained constant at 11%. Trainees recorded a higher number of cases of paediatric trauma than elective cases, particularly surgery for forearm, wrist and supracondylar fractures. Trainees infrequently performed surgery for lower limb trauma, emergencies such as musculoskeletal infection and paediatric elective procedures.

**Conclusions:**

Paediatric orthopaedics is an integral part of orthopaedic training. Our results suggest that paediatric orthopaedic experience at CCT may not satisfy the requirements of the JCST curriculum. Future curriculum adjustments and additional training methods may be required to ensure trainees obtain the necessary experience to meet both the JCST standards and the demands of paediatric trauma care.

## Introduction

In the United Kingdom (UK), trauma and orthopaedic surgeons must complete a paediatric attachment as part of their training as mandated by the Joint Committee on Surgical Training (JCST).^1^ The curriculum specifies a range of paediatric procedures that trainees should be competent to manage independently (level 4) upon qualification. Although a 6-month placement is recommended, a minimum of 3 months is permitted to accommodate programme delivery. This represents a limited amount of time to cover the breadth of paediatric orthopaedics. Training opportunities are further compounded by the limited operating time available to trainees, which has been further impacted by the COVID-19 pandemic.^2^

Getting It Right First Time (GIRFT) is a UK National Health Service (NHS) initiative that reviews medical and surgical specialties, assessing the state of current practice and how this can be improved.^3^ The Paediatric Trauma and Orthopaedic GIRFT report was published in April 2022, and outlines that consultant orthopaedic surgeons must have sufficient experience managing children’s orthopaedic conditions. The report highlights the importance of having adequately trained paediatric orthopaedic surgeons in specialist centres to facilitate appropriate care for patients that cannot be managed in peripheral hospitals.^4^

In considering the workforce, the GIRFT report highlights surgeons in training, recommending that training in paediatric orthopaedics should be maintained for all orthopaedic registrars to meet future workforce needs.

This study aimed to examine the paediatric operative exposure of UK trained orthopaedic registrars obtaining a Certificate of Completion of Training (CCT). It describes overall operative numbers, the proportion of paediatric cases, breakdown of trauma and elective surgeries, operative autonomy and experience in key procedures highlighted by the JCST curriculum. In quantifying the paediatric operative experience of trainees, the authors evaluate whether this exposure is sufficient to satisfy the requirements outlined by the JCST curriculum.

## Methods

### Study design

This was a national retrospective cohort study of all UK trainees holding a national training number (NTN) in trauma and orthopaedic surgery who completed their training between 1 January 2018 and 1 January 2024.

### Data sources

Specialty training (ST) in orthopaedics in the UK runs from ST3 to ST8, a 6-year training programme. This follows the completion of a 2-year core surgical training programme, or equivalent.

After selection trainees are enrolled by the JCST, who are also responsible for monitoring the trainees’ progress and recommending them to the UK General Medical Council for certification at the completion of training. A trainee’s progress is monitored via the Intercollegiate Surgical Curriculum Programme (ISCP), an online portfolio that houses the curriculum for the trainees’ surgical sub-specialty.^5^

A trainees’ operative experience is recorded via the eLogbook.^6^ This online platform allows the trainee to record operations they are involved in. In addition to the type of operation performed, an entry records patient demographics, urgency of the procedure and the level of supervision the trainee received. The level of supervision is recorded as: A, assisted; O, observed; STS, supervised-trainer scrubbed; STU, supervised – trainer unscrubbed but in theatre; P, performed; and T, training a trainee. Only operations coded as A, P, STS, STU or T count towards certification, referred to as indicative numbers. A minimum of 1,800 indicative cases are required for certification, with a set number of ‘index’ or ‘indicative’ cases required.^1^

The only paediatric index procedure is management of a displaced supracondylar fracture, but trainees may perform other index procedures such as intramedullary nailing, osteotomies, tendon repair, plate fixation tension band wiring and K-wiring, all of which are frequently encountered in managing paediatric patients.^1^ Furthermore, there are paediatric procedures outlined in the JCST curriculum that a trainee must be competent to manage independently (level 4), including: anterior drainage of a septic hip, in situ pinning of a slipped upper capital epiphysis and spica application or flexible nailing for femoral fractures.^1^

### Study population

To establish the study population, a data request was made to the JCST Data Analysis, Audit and Research Group (DAARG). JCST administrative records were used to identify all trainees in England, Wales, Scotland and Northern Ireland who achieved a CCT in orthopaedics between 1 January 2018 and 1 January 2024. Only trainees holding an NTN were included because the aim of this study was to assess the experience in paediatric orthopaedic surgery provided by UK orthopaedic training. The identified trainees’ operative records were obtained from the eLogbook. Each trainee was assigned an anonymous identifier to allow the combination of data. Regional data are not provided by DAARG and so only a national overview was possible.

To avoid confounding from operative experience obtained outside the training programme, only eLogbook entries from the start of a trainees’ training at ST3 to the end of their training at ST8 were utilised. To identify these dates, ISCP placement data were used for the previously identified trainees and applied as a filter to the eLogbook data output. The year 2018 was used as the start of the study window because prior to this placement data were incompletely recorded on the ISCP, as these trainees would have commenced training before 2012.

### Statistical analysis

Descriptive statistics were performed on all data. Before data output was created, visual assessment of normality with histograms and Q–Q plots was performed. The data were found to be non-normally distributed, and therefore data were presented as medians with interquartile ranges (IQR). Data were analysed to present yearly trends in overall operative experience, paediatric experience, operative autonomy in paediatric orthopaedics and experience of specific operative procedures in paediatric orthopaedics. Paediatric operations were defined as those performed in patients less than 17 years of age.

Specific procedures were broken down by level of supervision, with the STS and STU categories combined to indicate procedures a trainee has performed while being supervised, either directly or indirectly. The P and T categories were combined to indicate operations a trainee has performed independently without supervision. Elective cases were grouped into developmental dysplasia of the hip (DDH), congenital talipes equinovarus (CTEV) and cerebral palsy (CP) for analysis as per eLogbook categories. Trauma case operative codes were analysed individually. Anatomical sites where operative numbers were low and/or the logbook contains multiple codes were grouped together for analysis.

Data analysis was performed in Microsoft Excel (version 2308, Microsoft Corporation, USA).

### Ethics and consent

Part of the terms and conditions of the eLogbook that are accepted prior to usage include the use of anonymised data for research and analysis (section 11.3).^6^ All identified trainees were assigned an anonymous numerical code to avoid personal identification. Formal ethical approval was therefore not sought.

## Results

### Study population

**Figure 1:**
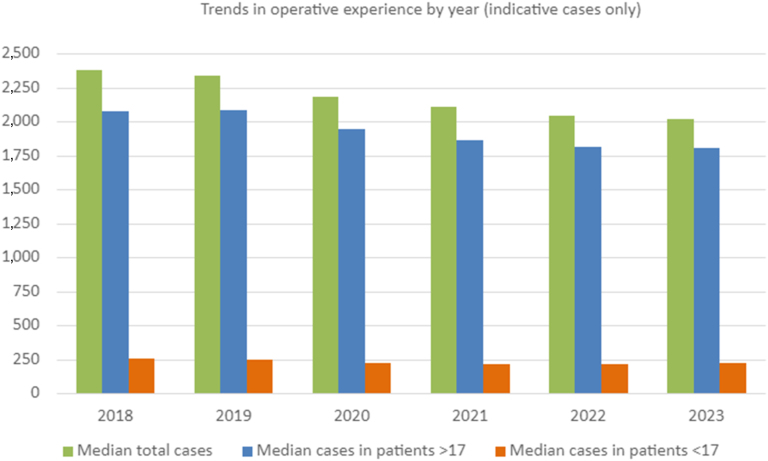
Trends in operative experience by year (indicative cases only)

Some 884 trainees were identified who achieved a CCT between 1 January 2018 and 1 January 2024, who had recorded a total 1,994,235 operative cases. The median total number of cases recorded per trainee logbook was 2,165 (IQR 1,960–2,452), and the median number of indicative cases was 2,162.5 (IQR 1,959–2,443; Table 1). There was a downward trend in the number of cases recorded in trainee logbooks across the study period (Figure 1).

**Table 1:** Total operative exposure to paediatric and adult orthopaedic surgery throughout higher specialty training (ST3–ST8)

Year of CCT	No. of trainees	Median total no. of cases [IQR]	Median no. of indicative cases [IQR]	Median no. of indicative cases <17 (%) [IQR]	Median no. of indicative cases >17 (%) [IQR]
2018	135	2,382 [2,161–2,718]	2,375 [2,156–2,715]	253 (10.7) [210–306]	2,077 (87.5) [1,881–2,390]
2019	123	2,338 [2,118–2,643]	2,336 [2,110–2,625]	248 (10.6) [186–329]	2,084 (89.2) [1,876–2,327]
2020	159	2,184 [1,988–2,490]	2,181 [1,986–2,483]	222 (10.2) [187–285]	1,941 (89.0) [1,759–2,248]
2021	125	2,109 [1,960–2,338]	2,104 [1,959–2,330]	218 (10.4) [181–272]	1,860 (88.4) [1,757–2,077]
2022	175	2,044 [1,918–2,308]	2,029 [1,918–2,301]	219 (10.8) [181–274]	1,812 (89.3) [1,704–2,047]
2023	167	2,015 [1,865–2,284]	2,015 [1,859–2,272]	224 (11.1) [186–280]	1,804 (89.5) [1,649–2,030]
Total	884	2,165 [1,960–2,452]	2,162.5 [1,959–2,443]	229 (10.6) [186–286]	1,923.5 (88.9) [1,743–2,187]

CCT = Certificate of Completion of Training; IQR = interquartile range

### Operative experience in paediatric orthopaedic surgery

Paediatric operations accounted for 10.6% of trainee operative experience at CCT during the study period (median 229 cases, IQR 186–286; Table 1). This percentage remained consistent across the study period despite an overall fall in total operative numbers. In comparison, adult operating accounted for 88.9% of operative experience at CCT (median 1,923.5 cases, IQR 1,743–2,187). Again, this percentage remained consistent over the study period.

**Figure 2:**
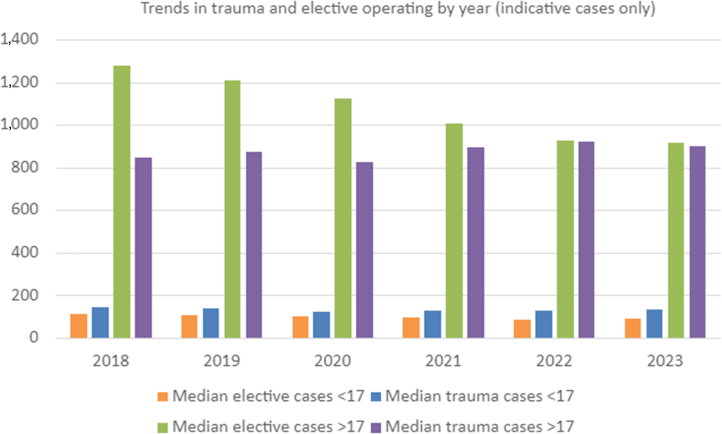
Trends in trauma and elective operating by year (indicative cases only)

Trainees performed more trauma cases in children (median 130.5 cases, IQR 107–159; Table 2) during their training than elective cases (median 96 cases, IQR 61–139). The reverse is true for adult operating. Trainees performed a median 879 trauma cases (IQR 762–1,012) during their training programme, compared with a median 1,047.5 elective cases (IQR 888–1,244). Elective case numbers followed a downward trend in both children and adults from 2018 to 2023. Trauma operating numbers also fell in children, but adult trauma operating numbers increased from 2018 to 2023 (Figure 2).

**Table 2:** Trauma/elective split in paediatric and adult orthopaedic surgery throughout higher specialty training (ST3–ST8, indicative numbers only)

Year of CCT	No. of trainees	Median no. of elective cases <17 [IQR]	Median no. of elective cases >17 [IQR]	Median no. of trauma cases <17 [IQR]	Median no. of trauma cases >17 [IQR]
2018	135	112 [82–157]	1,280 [1,049–1,483]	141 [110–169]	848 [736–1,010]
2019	123	105 [58–177]	1,207 [1,065–1,384]	137 [109–165]	875 [736–1,012]
2020	159	100 [68–142]	1,125 [978–1,320]	121 [102–156]	824 [716–989]
2021	125	94 [65–126]	1,004 [888–1,142]	129 [106–161]	892 [787–1,004]
2022	175	85 [54–132]	925 [818–1,056]	129 [111–158]	923 [785–1,048]
2023	167	89 [49–127]	914 [755–1,104]	134 [107–158]	897 [789–1,016]
Total	884	96 [61–139]	1,047.5 [888–1,244]	130.5 [107–159]	879 [762–1,012]

CCT = Certificate of Completion of Training; IQR = interquartile range

### Operative autonomy in paediatric orthopaedic surgery

Trainees recorded the majority of both elective and trauma procedures in children as supervised operating (55% STS/STU). Trainees performed more trauma (median cases 70, IQR 53–92; Table 3) than elective cases (median cases 48, IQR 27–73). Unsupervised operating (P/T) was uncommon in elective surgery, with a median of 5 cases (IQR 2–11) per trainee during their training. In comparison, a median of 33 unsupervised cases were recorded per trainee (IQR 19–49; Table 3) for trauma.

**Table 3:** Operative autonomy in children’s orthopaedic surgery throughout higher specialty training (ST3–ST8)

	Elective children’s orthopaedics – Median [IQR]			Trauma children’s orthopaedics – Median [IQR]		
Supervision level	O/A	STS/STU	P/T	O/A	STS/STU	P/T
Year of CCT						
2018	43 [29–73]	52 [35.5–72]	8 [4–17]	24 [16–36]	64 [43.5–83.5]	45 [27.5–67.5]
2019	40 [22.5–70.0]	50 [20–77]	7 [2–17.5]	24 [15–36]	69 [53–91.5]	38 [21–55]
2020	38 [24–60.5]	51 [31–79]	5 [2–10]	22 [13–33]	66 [50–89]	31 [16.5–47]
2021	35 [22–53]	47 [29–71]	5 [2–10]	23 [14–33]	66 [52–95]	32 [17–46]
2022	37 [17.5–58]	34 [27–70.5]	4 [1–9]	22 [14–32.5]	74 [55–95]	32 [17–45]
2023	32 [18.5–48.5]	32 [24.5–71]	3 [1–8]	22 [16–31.5]	76 [61–94]	28 [15–41.5]
Total	37 [22–60]	48 [27–73]	5 [2–11]	23 [15–33]	70 [53–92]	33 [19–49]

A = assisted; CCT = Certificate of Completion of Training; IQR = interquartile range; O = observed; P = performed; STS = supervised-trainer scrubbed; STU = supervised-trainer unscrubbed but in theatre; T = training a trainee

### Specific surgical case exposure

#### Elective surgery

Elective case exposure was grouped to cover three common paediatric conditions (DDH, CTEV and CP), because it was felt that recording individual procedures for these would yield low numbers (Table 4). The eLogbook also categorises specific operations into these groups when a trainee enters a case.

**Table 4:** Specific surgical case exposure (elective surgery) at completion of training

	No. of trainees who recorded procedures STS/STU	Supervision level (median cases in logbook per trainee) [IQR]			
	*n* (%)	O/A	STS/STU	P/T	Total
Operation group (as per elogbook operation groupings)					
Surgery for DDH	644 [72.9]	6 [2–11]	2 [0–5]	0 [0–0]	9 [4–16]
Surgery for CTEV	471 [53.3]	1 [0–4]	1 [0–3]	0 [0–0]	3 [1–7]
Surgery for CP	738 [83.5]	4 [1–8]	5 [1–12]	0 [0–0]	10 [4–21]

A = assisted; CP = cerebral palsy; CTEV = congenital talipes equinovarus; DDH = developmental dysplasia of the hip; IQR = interquartile range; O = observed; P = performed; STS = supervised-trainer scrubbed; STU = supervised-trainer unscrubbed but in theatre; T = training a trainee

Operations for CP were most recorded, with a median of ten total cases per trainee (all indicative supervision codes) at completion of training (IQR 4–21). Some 83.5% of trainees had performed an STS/STU operation for CP (median 5 cases, IQR 1–12); 72.9% of trainees had performed an STS/STU operation for DDH, with a median of 9 cases recorded (IQR 4–16), and 2 (IQR 0–5) performed STS/STU. Surgery for CTEV was the least recorded, with 53.3% of trainees having performed an STS/STU operation for CTEV, recording a median of 3 cases (IQR 1–7), and a median of 1 case STS/STU (IQR 0–3).

#### Trauma surgery

Trauma cases were analysed as specific operation codes, apart from certain anatomical sites where numbers were expected to be low, and the logbook contains multiple operation codes for a single site (e.g. lateral condyle fractures). The specific operations were chosen because they were felt to represent common paediatric trauma cases and emergencies that a non-paediatric orthopaedic surgeon may encounter as part of the acute take Table S1 (available online).

The most recorded operations were ‘Fracture distal radius MUA & POP (manipulation under anaesthesia & plaster of Paris)’ (median 17 cases, IQR 11–23; 97.9% had performed STS/STU), ‘Supracondylar/extra-articular distal humerus elbow fracture MUA ± percutaneous wires’ (median 12 cases, IQR 9–15; 99.3% had performed STS/STU) and ‘Fracture shaft radius MUA & POP’ (median 10 cases, IQR 7–14; 95.2% had performed STS/STU).

Although K-wiring for supracondylar fractures was commonly reported, application of traction for these injuries, which has now been added to the certification guidelines for trauma and orthopaedics, was rarely reported, with only 2% of trainees having performed this. This is in keeping with the results of the Supra Man Collaborative Study which found no cases of supracondylar fractures were managed with traction.^7^ However, both data sets may not capture cases managed with traction because they may not have a recorded surgical procedure.

Other elbow injuries were also rarely performed. Some 66.4% of trainees have performed an operation STS/STU for a lateral condyle injury when they complete their training, recording a median of 1 case STS/STU (IQR 0–2); 52.4% have performed an STS/STU operation for a radial neck fracture, recording a median of 1 case STS/STU (IQR 0–1).

Forearm and wrist injuries were commonly recorded as noted above. In addition to MUA and POP for wrist and forearm fractures, most trainees had performed TENS (titanium elastic nail) nailing of the radius (91.5%) STS/STU, and K-wiring of distal radius fractures (97.9%) STS/STU.

Lower limb trauma operative experience was less than that of the upper limb. The most recorded procedure was ‘Ankle fracture/dislocation ORIF (open reduction internal fixation)’ (median 3 cases, IQR 2–5; 84.0% had performed STS/STU).

Specific to the hip, 58.5% of trainees had pinned a slipped upper femoral epiphysis (SUFE) STS/STU, with a median of 1 case (IQR 0–2) STS/STU recorded. Only 23.6% of trainees had performed a septic hip washout in a child STS/STU during their training, with a median of cases 0 recorded (IQR 0–1).

Operations for femoral fractures were also infrequent. Some 57.8% of trainees had performed elastic nailing for a femoral fracture, with a median of 1 case (IQR 0–2) performed STS/STU recorded. Only 27.5% of trainees had applied a hip spica with a median of 0 cases (IQR 0–1) performed STS/STU recorded. Elastic nailing in the tibia was also uncommon, with 57.8% of trainees having performed this procedure STS/STU, with a median of 1 case (IQR 0–2) recorded STS/STU across all logbooks.

## Discussion

Throughout the study period, our data demonstrated that overall trainee operative numbers at CCT consistently decreased between 2018 (2,382 cases) and 2024 (2,015 cases). This decrease was also observed in total paediatric operative numbers; however, the proportion of paediatric cases remained stable at 10.6%. The ORTHOPOD Study reported that 10.7% of the UK trauma burden requiring operative intervention involved paediatric patients. Our findings align with this, highlighting that trainees obtain paediatric surgical experience across a range of departments, rather than being limited to their paediatric orthopaedic rotation.^8^

Several factors may explain the overall reduction in operative numbers observed. The decline in the traditional ‘firm’ structure and loss of the close trainee–trainer relationship that fostered an apprenticeship-style learning environment may have impacted training opportunities.^9^ The impact of the COVID-19 pandemic is likely a significant contributor to the reduction in both overall trainee operative numbers and exposure to paediatric cases for trainees within our cohort. The pandemic caused a widespread reduction in trauma presentations as public behaviour changed.^10^ Delivery of orthopaedic services was amended with cessation of elective practice and an increased threshold for surgical management where appropriate.^11^ In addition, many trainees were re-allocated during the initial phases of the pandemic. As a result, several trainees received an extension to their CCT date. ^12,13^

Although overall operative numbers decreased, the proportion of paediatric cases remained consistent. Trainees were exposed to more paediatric trauma than elective cases across all years examined. This is expected given the higher burden of paediatric trauma and simple fractures, which are managed across the trauma network. Trainees typically encounter paediatric trauma cases on operating lists at district general hospitals throughout training, whereas elective cases are managed in centres with a dedicated paediatric orthopaedic service. Conditions like DDH, CTEV or neuromuscular pathology requiring surgical intervention are relatively rare.^14–16^ As a result, elective paediatrics has increasingly moved towards tertiary referral centres, being managed by sub-specialist consultant paediatric trauma and orthopaedic surgeons.^4^

Examining operative autonomy, both paediatric elective and trauma cases were most coded as STS/STU, demonstrating that trainees are being trained in paediatric operating. As described, trauma made up most of trainees’ paediatric operative experience. Comparing operative autonomy between trauma and elective cases, trainees rarely performed elective procedures unsupervised (P/T), with a median of five cases across the cohort. Paediatric trauma saw a higher frequency of unsupervised procedures being undertaken by trainees, with a median of 33. This highlights the specialised nature of paediatric elective practice, which most trainees may not be required to perform in their consultant practice. Paediatric trauma is more frequently encountered, often comprising simple fractures and is a skill required of orthopaedic surgeons obtaining a CCT. Trainees looking to sub-specialise in paediatric orthopaedics will often undertake specialist post-CCT fellowships to further develop their skillset.

Elective paediatric cases are subcategorised by the eLogbook into the more common elective conditions of DDH, CP or CTEV. The limited experience in managing these conditions experienced by trainees aligns with the recommendations of the GIRFT report that such cases should be undertaken in a specialist centre by dedicated paediatric orthopaedic surgeons.^4^ Despite this, these cases are an opportunity to develop transferrable skills and should remain an integral part of orthopaedic training.

In the breakdown of trauma cases, upper limb fractures were most commonly encountered by trainees, including: ‘Fracture distal radius MUA & POP’, ‘Supracondylar/extra-articular distal humerus elbow fracture MUA ± percutaneous wires’ and ‘Fracture shaft radius MUA & POP’. Most trainees recorded these procedures STS/STU or P, which aligns with the most frequently reported paediatric trauma requiring surgical intervention in the literature.^9^ Many trainees also recorded having performed K-wires, ORIF or TENS nailing for wrist and forearm injuries. Management of supracondylar fractures represents an index procedure required by the curriculum, with a minimum of five cases to be performed. Our results demonstrate a median case volume of eight STS/STU per trainee, demonstrating that indicative cases are likely sought out by trainees to meet curriculum requirements.

Lower limb fractures were less frequently recorded than upper limb injuries. The most coded operation for lower limb injuries was ‘Ankle fracture/dislocation ORIF’. Procedures involving femoral and tibial fractures, such as elastic nailing, were rarely recorded, with a median of just one case. In addition, only 27.5% of trainees report having applied a hip spica for a femoral fracture during their training, and 58.5% had performed cannulated screws for a SUFE, with a median of one STS/STU case.

Given the rarity of SUFE cases and the limited exposure trainees receive during their training, general orthopaedic surgeons are not best placed to manage SUFE cases without input from paediatric orthopaedic specialists. Similarly, open washouts of hip and knee joints were encountered infrequently by trainees with 23.6% (hip) and 22.6% (knee) having recorded this procedure STS/STU. This raises a similar question about whether general orthopaedic surgeons are appropriately trained to safely manage musculoskeletal infection in paediatric patients.

The JCST curriculum outlines the training requirements necessary for a day one orthopaedic consultant to safely manage the unselected emergency take.^1^ It specifies that, upon achieving CCT, trainees must be competent in performing an anterior drainage of a septic hip, in situ pinning of a SUFE and spica application or flexible nailing of a femoral fracture, including managing common complications. However, as highlighted in our results, the median case load for these procedures ranges between 0 and 1, indicating an inexperience in managing these cases. For non-emergent low-volume cases such as a SUFE, establishing locally agreed referral pathways to tertiary centres would be pragmatic to ensure optimal patient management in line with GIRFT principles. For infrequently encountered emergencies such as a septic hip washout, relying on training numbers is insufficient and alternative training methods such as simulation, cadaveric workshops and additional courses might be beneficial in ensuring trainees have the necessary competence.^17^

### Study limitations

Regarding limitations, eLogbook data are reliant on the accuracy with which a trainee records their operative cases. In a recent national survey of UK surgical trainees, inaccurate operative recording was widely reported, and was largely related to trainees feeling pressure to achieve operative numbers to progress in training.^18^ Despite this, we feel our results provide a useful insight into the current experience trainees receive in children’s orthopaedics. It is also important to acknowledge that the spectrum of procedures for management of paediatric conditions is not exclusively captured by theatre cases recorded in the eLogbook; for instance, some procedures such as percutaneous tendo-Achilles release may be performed in a clinic setting. Furthermore, some procedures may have been recorded under a non-paediatric logbook code.

Finally, although our data highlight that trainees may not meet the requirements of the JCST curriculum, it must be recognised that the operative numbers seen probably represent the limit of experience that can be achieved within a single 6-month placement. If further competencies are required, then trainees may require further time in paediatric orthopaedics, or training through alternative methods as mentioned previously. We would advocate as a minimum that paediatric placements are 6 months in length.

## Conclusions

The JCST orthopaedic curriculum sets out clear expectations for trainees to develop competence in managing paediatric orthopaedic conditions independently. However, our data suggest that the paediatric experience gained by trainees at CCT may not satisfy these requirements.

Key procedures deemed essential for independent practice are rarely encountered during training, making it unrealistic to expect trainees to be fully competent in managing these conditions. Our findings highlight the need for curriculum adjustments to ensure trainees gain the necessary experience to meet both JCST standards and the demands of paediatric trauma care.

Maintaining high-quality paediatric orthopaedic training is essential, not only to equip future consultants with the skills to manage paediatric patients effectively, but also to inspire the next generation of paediatric orthopaedic surgeons.
